# Notes of a Case of Uterine Polypus Terminating in Fatal Sphacelation

**Published:** 1816-12

**Authors:** Richard Bird

**Affiliations:** Jun. Surgeon


Harwich, October 10, 1816.

---

### *Notes of a Case of Uterine Polypus terminating in fatal Sphacelation.* By RICHARD BIRD, Jun. Surgeon.

OCTOBER 13th, 1816. On examination of the body of a woman, aged about 33, in the Poor-house of Fazeley, this morning by myself and Dr. Palmer, the following appearances were noted. She had been dead but a few hours.

*External View.* The expresssion of the face was nearly the same as it had been before death, on the preceding night. The cheeks had a puffy appearance, and their cellular membrane, like that of every other part of the body, evidently contained an unusual quantity of serous fluid. The parietes of the hypogastric and umbilical regions were elevated by a tumour, clearly consisting, from its figure, of the distended bladder. A large, rounded, bloody mass, in a state of sphacelation, and tightly embraced above by the vagina, protruded externally from between the labia pudendi. The latter were, in several places, ulcerated from its pressure.

*Abdomen.* Much fat was accumulated in the cellular structure. The bladder contained upwards of three pints of healthy-looking urine; and exhibited considerable turgescence of its vessels without any mark of decided inflammation: its ureters were much distended: the kidnies sound. After the urine had been evacuated, and the bladder allowed to collapse, the uterus was found much enlarged, and its cavity evidently occupied by a firm unyielding substance, which might be traced in its descent beneath the pubic arch, and was decidedly continuous with the external tumour. Finding it impossible, from the bulk of the protruded portion, to retract it into the pelvis, and equally so to draw out all the viscera of this cavity without disfiguring the external parts, we were under the necessity of cutting transversely through the womb and contained polypus, just above the cervix of the former. This done, the external and lower portion of the polypus came away without difficulty; while that, adherent to the uterus, with the whole of the pelvic organs, was removed, in the ordinary manner, for closer examination. The uterus was about five times the size of the organ in its unimpregnated state; its parietes about one-third of an inch in thickness. The whole of its cavity was occupied by the base of the polypus, which arose exactly at the fundus, and would have measured at least three inches in diameter. From this part, the diseased mass was somewhat diminished in size until it had passed the os externum; when it again expanded into an oblong, irregularly rounded form.* The structure of the whole was firm, dense, and unyeilding. Decomposition had commenced only on the surface of the protruded part. There were some marks of inflammation of the uterine membrane around the base of the polypus: in other respects, the organ itself was sound. The ligamenta lata were smaller than natural, from the increased volume of the womb. The ovaries were unusually large and firm, and displayed several corpora lutea. The fimbriated extremities of the Fallopian tubes had a highly vascular, and even bloody appearance. The colon contained a large accumulation of indurated fæces. The whole track of the small intestines was of a dusky colour; and the mesenteric blood-vessels very much loaded. The pancreas, in its internal structure, presented a bright pinkish colour, and conveyed a rough and coarse sensation to the finger, as though its component granules were harder and larger than natural: it had evidently been the seat of increased action. The liver (with the exception of the gall-bladder, which was small, thickened in its coats, and shewed a strongly-marked contraction near its fundus) the stomach and spleen were in a natural condition. But there existed a singular disproportion in the size of the two main vascular trunks of the abdomen. While the inferior vena cava and its branches were extraordinarily large, the abdominal aorta by no means equalled in circumference one of the renal veins; nor was its inferior mesenteric branch more capacious than a crow-quill. The abdominal viscera, probably in consequence of the pressure of the distended bladder and uterus, had thrust upwards the diaphragm and encroached in an extraordinary

---

* The length of the whole polypus, from the base to the extreme point, was eight inches, after it had been, for some days, immersed in strong solution of muriate of quicksilver; and the longest diameter of the apex must have considerably exceeded that of the base.

degree upon the cavity of the chest; the organs of which, excepting some adhesions of the pleuræ, were quite healthy. The brain was not examined.

---

The subject of this case was a woman of very loose and profligate character. She had borne three children, and twice or thrice suffered abortion. Her last delivery, which took place about a year and a half since, was attended with extraordinary difficulty. Sometime after this, she first began to complain of severe pains in the region of the uterus, and of a pale, glairy discharge from the vagina. The symptoms were supposed to arise from some organic disease, probably scirrhus of the womb; but the woman did not submit to the examination which could alone accurately determine this point. Three nights previously to her death, she walked to Tamworth; and, on her return, the tumour first shewed itself externally. Upon reaching home, she complained of excruciating pain, and seemed to experience great difficulty in voiding her urine. I was, at that time, absent from Tamworth, and did not see her till Saturday, the twelfth. She was then evidently dying. The pulse was extinct; the extremities cold; articulation indistinct. A vomiting, with which she had been affected, had ceased; but subsultus tendinum was present. The mass projecting from the vagina was in a black, fœtid, gangrenous condition. In fact, the case exhibited all the ordinary symptoms of advanced and fatal typhus. The fæces and urine were said to have been evacuated with tolerable freedom. Every attempt even at palliation was now obviously hopeless. The poor woman died about four o'clock on the morning of the following day.

Had the precise nature of the case been ascertained by examination *par vaginam* before death, the extraordinary breadth of the base of the polypus would, I conceive, have precluded every well-grounded expectation of a fortunate issue from any attempt at removal. It is probable, from the unusual difficulties attendant on the last delivery, that the process of morbid alteration had, at that period, commenced in the membranous lining of the uterus.

*Tamworth, October* 30, 1816.

